# A Clinical Re-Audit (Cycle 1) on the Monitoring and Management of Antipsychotic-Induced Hyperprolactinaemia

**DOI:** 10.1192/bjo.2025.10620

**Published:** 2025-06-20

**Authors:** Noman Khan, Robert Orange

**Affiliations:** South West Yorkshire NHS Foundation Trust, Dewsbury, United Kingdom

## Abstract

**Aims:** Antipsychotic medications are one of the major iatrogenic causes of hyperprolactinaemia with the attendant short- and long-term effects and risks associated with it. The audit sought to answer the question: are we monitoring for and managing hyperprolactinaemia caused by our medications appropriately, and to compare the results with previous audit.

**Methods:** A literature search for relevant data and standards with regards to monitoring and management of hyperprolactinaemia was conducted.

The audit was based on the standards derived from South West Yorkshire NHS Partnership Foundation Trust’s (SWYPFT) standards, NICE guidelines, and the Maudsley Prescribing Guidelines In Psychiatry (14th edition), focusing on the Trust’s standards.

The total population under consideration included every patient under the care of the North Kirklees, Community Mental Health Team (CMHT), Older people services, in the time period between 16 January 2024 and 15 August 2024, who was using antipsychotic medication.

Results:

The total number of patients in the study was 78.

1. Patients not requiring monitoring (n=35). These patients were on medications such as olanzapine, quetiapine, or aripiprazole and did not require monitoring.

2. Patients requiring monitoring (n=43). Out of the 43 patients:

First-time antipsychotic users (n=15): 10 patients did not have their prolactin monitored; 5 patients had their prolactin monitored.

Long-acting injection patients (n=8):6 patients had their prolactin monitored; 2 patients did not have their prolactin monitored.

Oral antipsychotic medication patients (n=20):11 patients had their prolactin monitored; 9 patients did not have their prolactin monitored.

Monitoring outcomes:

Out of the 43 patients requiring monitoring, 22 patients had their prolactin monitored (51%).

21 patients (49%) did not have their prolactin monitored.

**Conclusion:** As compared with the first audit, this re-audit showed only a 10% increase (from 41% to 51%) in monitoring of prolactin levels, still far away from achieving the 100% monitoring goal.

Perhaps, big improvement was not seen because of clinical perception of less significance of prolactin level below 2500 in old age patients.

